# Suction-Gas Plant *v*. Boiler Engine

**Published:** 1912-04-27

**Authors:** Wm. Kay

**Affiliations:** Chorley Street, Bolton


### SUCTION-GAS PLANT *v.* BOILER ENGINE.

*To the Editor of* The Hospital.

Sir,—In addition to our local infirmary we are building a nurses' home for forty beds, and we are desirous of making heating arrangements for all the premises. If any of your readers connected with any institution of a similar nature can give any information respecting the installation of a suction-gas plant in place of boiler engine, &c., which I am informed will not cost half of the present boiler engine system, I should be very glad to be able to get to some authority on this matter through your paper.— Yours respectfully,

Wm. Kay.

Chorley Street, Bolton, April 18.

